# Prescriptions and Their Owners

**Published:** 1891-04-25

**Authors:** 


### The Hospital

A WEEKLY JOURNAL OF

**Science, Medicine, Nursing, and Philanthropy.**

*For Hospitals, Asylums, Infirmaries, Dispensaries, Medical Practitioners, Students, Nurses, and the Charitable Public.*

VOL. X.—No. 239.] [APRIL 25, 1891.

### PRESCRIPTIONS AND THEIR OWNERS.

THE progress of evolution is converting the apothecary into the family physician. It is a common ambition among the ablest and most self-respecting of the younger practitioners to secure either a hospital appointment with a consultant's position, or a non-dispensing family practice. The man who keeps a surgery, or, more properly, a dispensary, does not usually secure such liberal fees as his non-dispensing neighbour; and there is, consequently, a tendency for him to sink to a less dignified level. It cannot be too emphatically protested, however, that true dignity does not consist in well-furnished consulting-rooms, or in the power to charge high fees, but in honourable conduct and sound professional capacity. Many a man who mixes his own medicines, and labels and wraps up the bottles after a hard day's professional visiting, has much more real merit and true dignity of character than some who hold hospital appointments and demand and receive the recognised two-guinea fee. This is but the statement of a common-place, we are aware; but it is very necessary sometimes to tell a man of undoubted honour that he is honourable, and a man of real worth that he is worthy. Merit, which is persistently unrecognised, may droop and wither for the want of recognition, just as a flower or a tree may shrivel and die when deprived of the light and warmth of the sun.

The general tendency of the medical profession towards higher culture and greater professional capacity brings about changes in the methods of conducting the daily business of practice. The son of the father who used to send his patient a bottle of physic after his visit, writes a prescription at the patient's house, and the latter gets the physic for himself at the neighbouring chemist's. From a medical point of view this change of procedure has its advantages and its disadvantages, and the same may be said from the patient's standpoint. One advantage secured to the doctor is the saving of labour and the diminution of expense. He charges for his visit, and he avoids both the cost and the trouble of preparing the medicine. A disadvantage is, that when once a patient has got possession of a prescription there is nothing to prevent him using it, again and again, without further consultation with the doctor. The latter is thus liable to the loss of a good many visiting or consulting fees.

All classes of physicians have long felt that the honour and glory of consulting practice had its drawbacks, and that one of those drawbacks was the giving of prescriptions over which no professional control was retained. In the case of the older consultants, whose fees range from two guineas upwards for each consultation, and who can always command full consulting rooms, this is a matter of comparatively small importance; but the question assumes a very different complexion when the family physician receives the small fee of five shillings for a professional visit, and gives up to the patient for his unrestrained use a prescription which may run for a week, a month, a year, or a lifetime. It is hardly surprising that under these circumstances some rather sharp notes of alarm should have been sounded in the professional journals, nor is it very strange that those notes of alarm should have been followed by suggestions indicative rather of fear than of wisdom. One family physician proposes to claim an absolute medical proprietorship in prescriptions. "My prescriptions are mine," says he, "and I mean to stick to them." He has been driven to this bold attitude of self defence by the discovery that one of his lady patients lent a particular prescription of his to eight other ladies, and so, as he puts it, practically robbed him of eight fees. Another family physician becomes quite Jesuitically hair-splitting in his attempt to deal with the difficult and complicated question. "My own opinion," he says, "is, and always has been, that the prescription does not legally belong to any of the parties concerned, but should be destroyed after it has fulfilled its mission." The idea of a prescription "fulfilling its mission," is distinctly poetical and original. This gentleman continues—"We pay for a railway ticket and it is taken from us on completing the journey. The prescription is nothing more than a convenient missive"—"missive" is good—"between doctor and chemist, and probably would not be entrusted to the patient if we had any other more convenient way of communicating our order. We do not address the prescription to the patient; we advise the chemist how he has to compound a certain number of ingredients for the patient; this being done, the prescription should be destroyed or returned to the 'author.'"

Now all this is exceedingly interesting and instructive to the student of psychology, as that science asserts itself in the medical mind. It shows how the "natural man" still exercises a dominating influence over the professionally differentiated specialist. The first doctor stands forth with all the truculence of a medical Dick Turpin, and insists that his prescriptions shall be returned to him on pain of—we know not what. The second glides into the subject with insinuating logic, and shows how proper and natural it is that the doctor, who is the "author" of a prescription, shall retain all the powers of copyright in his own work. It is a pity that patients do not occasionally read professional journals. The discussion would have been greatly improved, from the point of view of general edification, if one or two patients had added their contributions. Arguments are mostly inconclusive which discuss only one side of the question.

It seems to us that the interests of patient and doctor are not opposed to each other in this matter, but are identical. On the one hand, those doctors who wish "both to eat their cake and have it," make a mistake; and on the other, those patients who use a presciption for a longer time than it is ordered to be used, or who give it to their friends, make a still greater mistake. As a matter of fact, where proper relations subsist between doctor and patient, it is probable that very little injustice is done to the former. One thing seems to be quite certain, and that is, that the doctor who gives a prescription to his patient cannot both give it and keep it. All he can do is to instruct the sick person how long to take the medicine prescribed. If the latter continues to take it longer, he is exceedingly foolish. No honourable doctor ever thinks of limiting the time during which a prescription may be used merely for the sake of getting an additional consultation fee out of his patient. Any patient who has good reason to believe that his doctor is keeping him an unnecessary length of time on his visiting or consulting list ought to seek another doctor at once; and any doctor who asks his patient to return to his consulting room when there is no necessity for further consultation, is a deliberate cheat, and deserves all the contempt and obloquy which can be poured upon him. The physician, including of course, under this term, every member of the profession, occupies a position of peculiar delicacy towards his patient. The patient is ignorant, the physician has knowledge; the patient is fearful, the physician has the confidence of experience. What kind of doctor is he then who takes advantage of his patient's trustfulness to worry his mind with fear, and to extract from his pocket unnecessary fees? He is a scoundrel.

But on the other hand, whilst physicians must be as honourable as Cæsar's wife, because their position is one of such unlimited freedom, patients must not forget that he who dispenses honour and justice with his prescriptions, is entitled to honour and justice in return. Wise men and women clients will use a remedy for the exact period for which it has been prescribed. If they use it a very little longer the reasonable physician will not object; and they will probably not do themselves any great harm. But they should remember that in using a prescription longer than the specified time, there is always a possibility of their doing a serious injury to their own health; and this injury to their health is an injury and an injustice to the physician; not, be it noted, in the mere loss of one or two paltry fees, but in the wound it may inflict upon his reputation and his feelings in that his misused prescription has been a source of injury rather than of benefit to his patient.

Should patients ever give medical prescriptions to other persons? As a rule, certainly not. They may do untold injury by such acts. This is not said to frighten them; it is plainly and simply true. Modern remedies are of such a kind that they ought only to be handled by experts. The subject is of much importance, and space forbids us to expound the patients' aspect of the case as fully as it demands. But this is to be insisted upon: that if a high sense of duty compels the doctor to shrink with disgust from taking the least advantage of his patient, the very same sense of duty should compel the patient to make it a matter of conscience to avoid doing any kind of injury to the doctor.

